# Variation of Runoff and Precipitation in the Hekou-Longmen Region of the Yellow River Based on Elasticity Analysis

**DOI:** 10.1155/2014/929858

**Published:** 2014-05-12

**Authors:** Erhui Li, Xingmin Mu, Guangju Zhao, Peng Gao, Hongbo Shao

**Affiliations:** ^1^College of Water Resources and Architectural Engineering, Northwest A&F University, Yangling, Shaanxi 712100, China; ^2^Institute of Soil and Water Conservation, Northwest A&F University, Yangling, Shaanxi 712100, China; ^3^Institute of Soil and Water Conservation, Chinese Academy of Science and Ministry of Water Resources, Yangling, Shaanxi 712100, China; ^4^Key Laboratory of Coastal Biology & Bioresources Utilization, Yantai Institute of Coastal Zone Research (YIC), Chinese Academy of Sciences (CAS), Yantai 264003, China

## Abstract

Precipitation is very important to the formation of runoff, and studying of runoff variation and its response to precipitation has practical significance to sustainable utilization of water resources. The study used Mann-Kendall test, anomaly accumulation method, and precipitation elasticity of runoff method to analyze the changes in the relation of precipitation and runoff and the contribution of precipitation to runoff change in the Hekou-Longmen region (from 1957 to 2010), Huangfuchuan watershed (from 1954 to 2010), and Yanhe watershed (from 1952 to 2010) in the middle reaches of the Yellow River. The results showed that runoff appeared a significant decreasing trend (*P* = 0.01) while it was not significant in precipitation in all study areas. In particular, the reductions of average annual runoff in the Hekou-Longmen region, Huangfuchuan watershed, and Yanhe watershed were 72.7%, 87.5%, and 32.2%, respectively, during 2000–2010 compared to the 1950s. There existed two abrupt change points of the runoff in the Hekou-Longmen region and Huangfuchuan watershed, which were detected in 1979 and 1998. But in the Yanhe watershed only one abrupt change point was found in 1996. The precipitation elasticities of runoff were 1.11, 1.09, and 1.26, respectively, and the contributions of precipitation on runoff reduction were 26.4%, 17.9%, and 31.6%, respectively, in the Hekou-Longmen region, Huangfuchuan watershed, and Yanhe watershed.

## 1. Introduction

The current global climate change has changed the situation of water cycle, which causes a series of problems about water resources [1–3]. The temporal and spatial variation of runoff is an important part of hydrological cycle, which not only influence the exploitation and utilization of water resources but also influence the development of social economy.

The effect of climate change in runoff is a hot topic in hydrologic study of the world—for example, Asia [4–7], Africa [8–10], Europe [11–13], America [14, 15], and Australia [16–18]. Among these studies, the methods of regression analysis and hydrology model are applied popularly [19–23]. Ma et al. [24] used geomorphology-based hydrological model to conduct a quantitative assessment of the impact of climate variability and the indirect impact of human activity on the inflow into the Miyun reservoir of Beijing. Simulation results of the hydrological model showed that climate impact was accountable for about 55% of the decrease in reservoir inflow. Sun et al. [25] showed that runoff of Jinjiang basin in China exhibited an increasing trend in summer to early autumn, while in the remaining periods of a year it shows a decreasing trend analyzed by the hydrological model. Though hydrological physical process revealed by hydrology model method is relatively close to objective world, large numbers of observation data including precipitation, temperature, wind speed, and relative humidity are needed and calibration of model parameter can directly influence the accuracy of the results [26]. Statistical regression analysis is an easy method to use the observed data with long time series to build the statistical relation between climate and runoff, which is a quick method to evaluate the influence of climate change to runoff.

Climate elasticity of runoff is the most applied method in statistical regression analysis. The concept of climate elasticity was put forward by Schaake [27] to evaluate the influence of climate change to runoff. When precipitation elasticity of runoff is 1.5, which means the runoff changes 1.5% when the precipitation changes 1%. Nash and Gleick [28] studied the sensitivity of runoff to climate of Colorado River and showed that, when the temperature and potential evaporation keep unchanged, 10% increase of precipitation will cause 11% increase of runoff. Chiew [29] calculated precipitation elasticity of runoff index in 219 watersheds of Australia; the result varies from 2.0 to 3.5. Fu et al. [30] extended the single parameter precipitation elasticity of runoff index into a two-parameter climate elasticity index in two large watersheds. The results reflected the complicated nonlinear relationship among runoff, precipitation, and temperature. Hu et al. [31] calculated the contribution rates of climate change to runoff in upstream of Tanghe river in Baiyangdian basin using climate elasticity of runoff and hydrological simulation methods; the contribution rates were 40% and 38%, respectively. There was little difference between the two methods in calculating the contribution rates of climate change to runoff. The climate elasticity of runoff method has no consideration on the distribution and frequency of precipitation and on the change in underlying surface, so it only was used to evaluate the sensitivity of runoff on climate without or with little human disturbance. The result of climate elasticity of runoff did not appear much different from the result obtained from model simulation, which offer an easy way to calculate the sensitivity of runoff to precipitation in a long sequence. The Hekou-Longmen region (from Hekouzhen to Longmen hydrologic station) is located in the arid and semiarid areas, which is in the middle reaches of the Yellow River. The distribution of precipitation is uneven there. Since the precipitation is a direct natural factor of influencing runoff, the study on variation characteristics of precipitation and runoff has a great significance on management of water resources in river basin.

To address the impacts of climate change to runoff, the objectives of this study were (i) to explore the temporal trends of precipitation and runoff, (ii) to analyze the abrupt change point of runoff, and (iii) to estimate the influence of precipitation elasticity of runoff in Hekou-Longmen region of the Yellow River, China.

## 2. Study Area

**Figure 1:**
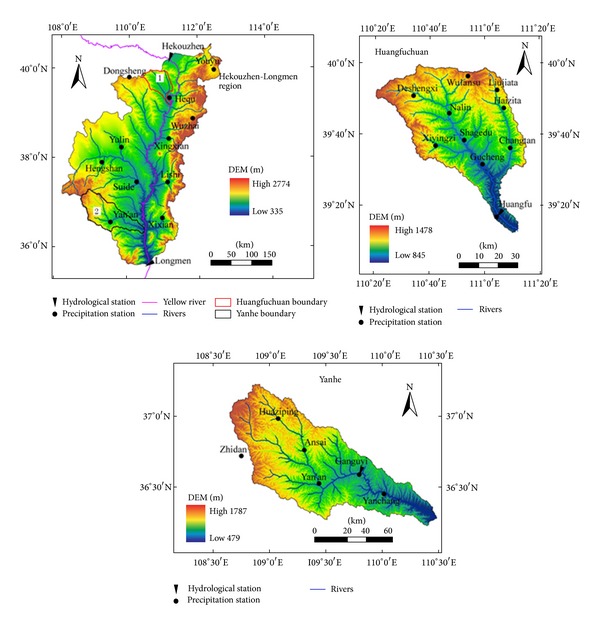
Location of the study area, precipitation stations, and hydrological stations.

The study areas are Hekou-Longmen region and the typical tributaries of the middle reaches of the Yellow River—Huangfuchuan and Yanhe (Figure 1). They have different land types and distribution of precipitation. The characteristics are detailed as follows.

Hekou-Longmen region is located in the upper middle reaches of the Yellow River at 108°02′E~112°44′E and 35°40′N~40°34′N with a catchment area of 111586 km^2^, which occupies 14.8% of total Yellow River basin. Tributaries in Hekou-Longmen region are very rich and there are 21 large tributaries flowing directly into the Yellow River. In this region, loess area occupies about 62%, windy desert area 24%, and bed-rock exposed area 14%. This area has loose soil, breaking terrain, crossing gully, and very sparse vegetation. What is worse, the rainfall is intense and concentrated, so soil and water loss is a serious problem in this area. The annual average precipitation is from 310 mm to 580 mm with an uneven spatial distribution.

Huangfuchuan basin is located in the north of Hekou-Longmen region at 110°18′E~111°12′E and 39°12′N~39°54′N with the river length of 137 km, and the catchment area is 3246 km^2^. The annual precipitation is 350 mm~450 mm, and more than 80% of it comes from June to September. There are complex geomorphological types including feldspathic sandstone hilly-gully region, the loess hilly-gully region, and sanded loess hilly-gully region. Vegetation in this area has been destroyed, meanwhile soil and water loss caused by the big terrain height difference and strong rainfall is severe, annually, which brought about 0.15 × 10^9^ t sediment load into the Yellow River, leading to a devastating impact on ecological environment and agriculture condition [32].

Yanhe River is located in the south of Hekou-Longmen region at 108°45′E~110°28′E and 36°23′N~37°17′N with a catchment area of 7687 km^2^. Annual precipitation is about 500 mm and average annual temperature is about 9°C in this area. From southeast to northwest, climate and temperature have an obvious gradient change. Vegetations from south to north, with the obvious change of environment gradient, are forest, forest and grassland, and grassland in turn [33]. In Yanhe basin, the loess hilly-gully region accounts for 90% of the total basin, and the slope in most areas is more than 15°, where the soil erosion is very serious. Vegetation and soil types have a significant difference in Yanhe and Huangfuchuan, so it is necessary to study runoff characteristics separately on the above three regions.

## 3. Data Sets and Methods

### 3.1. Data Sets

Considering the continuity and integrity of data, annual precipitation data during the period 1957–2010 were selected from China meteorological data sharing service System (http://cdc.cma.gov.cn/) with 11 well-distributed meteorological stations in Hekou-Longmen region (Figure 1). The average precipitation on the basin was interpolated by the method of Thiessen polygon. Annual runoff data of the Hekou-Longmen region during the period 1957–2010 were calculated by the runoff measurement data difference between Hekouzhen station and Longmen station. All the runoff data come from hydrological yearbook and water resources bulletin of Yellow River.

Annual runoff data of Huangfuchuan basin during the period 1954–2010 were collected from the Huangfu hydrological station with a controlled area of 3175 km^2^, accounting for 97.8% of basin area. Average precipitation of the basin was interpolated by the method of Thiessen polygon with the measured rainfall data of 10 well-distributed rainfall stations during the period 1954–2010 (Figure 1). The data of 1954–1989 were provided by hydrological yearbook of Yellow River, and the data of 1990–2010 were taken from the Yellow River conservancy commission.

Annual precipitation data of Yanhe basin were taken from annual data of hydrological stations in Yan'an (1952–2010), Ansai (1956–2010), Gongyi (1952–2010), Huaziping (1953–2010), and near river Zhidan (1956–2010). To make sure of the unity of data length, data of Yan'an station were adopted to interpolate the historical data of other stations, and the average precipitation of 6 stations during 1952–2010 were obtained by the method of Thiessen polygon. Annual runoff data during the period 1952–2010 were collected from Gongyi hydrological station with a control area of 5891 km^2^, accounting for 76.3% of basin area. Runoff data were all taken from hydrological yearbook of Yellow River.

### 3.2. Analysis Methods

Mann-Kendall (MK) test and anomaly accumulation method are applied to analyze the precipitation and runoff changes in the middle reaches of the Yellow River. The influence degree of runoff on precipitation change without the influence of human activities can be revealed by precipitation elasticity of runoff. The MK test has been widely used for assessing the significance trends in hydrological time series due to its robustness for nonnormally distributed and censored data [34–36]. The anomaly accumulation method is used in analysis of stage changes in hydrological time series [22]. The MK test and anomaly accumulation method will not be discussed here because they had been described in detail in many studies [37–39].

Climate elasticity of runoff is widely applied to quantify the sensitivity of runoff to climate parameters [40]. Climate elasticity of runoff may be defined as the proportional change in runoff, *R*, to the change in a climatic variable such as precipitation, *P*. Thus precipitation elasticity of runoff is defined as [27]

(1) $$\begin{array}{c} \varepsilon_{p}\left(P,R\right)=\frac{\partial R}{\partial P}\frac{P}{R}. \end{array}$$

A difficulty with estimation of elasticity is that it is often estimated from the partial differential of hydroclimatic variables but the distribution of hydroclimatic variables is always unknown. In order to resolve these problems, Sankarasubramanian et al. [41] introduced the nonparametric estimator:

(2) $$\begin{array}{c} \varepsilon_{p}=\text{median}\left(\frac{R_{i}-\bar{R}}{P_{i}-\bar{P}}\frac{\bar{P}}{\bar{R}}\right), \end{array}$$

where *R*_*i*_ and *P*_*i*_ are the runoff depth and precipitation (mm) of the *i*th year, respectively; $\bar{R}$ and $\bar{P}$ are the mean value of the runoff depth and precipitation (mm); *ε*_*p*_ is the precipitation elasticity of runoff.

## 4. Results and Discussion

### 4.1. Trend of Precipitation and Runoff

**Figure 2:**
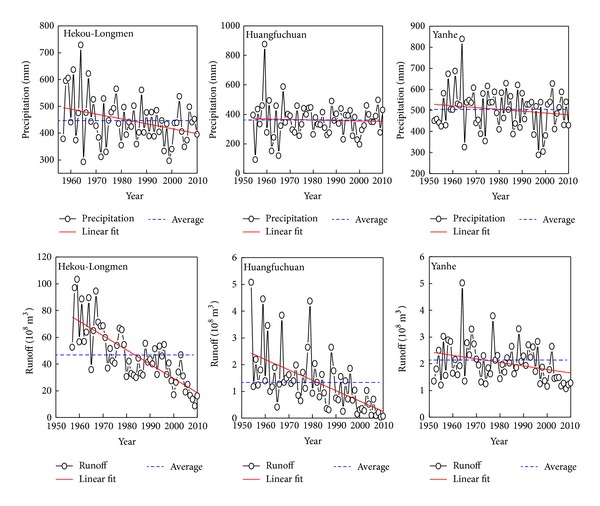
Temporal changes of precipitation and runoff in the Hekou-Longmen region, Huangfuchuan, and Yanhe.

According to the trend of precipitation and runoff in the Hekou-Longmen region, Huangfuchuan basin, and Yanhe basin (Figure 2), annual precipitation shows fluctuating changes and the annual runoff is in a decreasing trend. Before 1980s, annual runoff in the Hekou-Longmen region and Huangfuchuan is higher than the average value for many years and its variation is large. But after 1980s, the runoff is lower with a small variation, and, especially after the year of 2000, annual average runoff change appears a decreasing trend. However, the runoff variation in Yanhe River is relatively even, fluctuating up and down around the average value. After the year of 2000, runoff change also shows a sustainable decreasing trend.

Characteristics of precipitation and runoff in different decades in the Hekou-Longmen region, Huangfuchuan basin, and Yanhe basin are illustrated in Table 1. Precipitation amount in the 1950s and 1960s in the Hekou-Longmen region is abundant but becomes lesser in the 1990s. After 1980, the variation coefficient of precipitation tends to be stable with the value 0.14~0.15. The variation coefficient of runoff is the biggest at the beginning of the 21st century, which shows that the runoff in this period has a dramatic change and is greatly influenced by human activities. Precipitation amount in the 1950s in Huangfuchuan basin is the largest. Meanwhile, precipitation in the 21st century equated with the average annual one, but the runoff in the same period is about 1.0 × 10^8^ m^3^ lower than the average one. This phenomenon shows that the change of precipitation does not coincide with the runoff change, which may results from the large-scale returning farmland to forest (or grass), construction of check dams, and so on. Precipitation in Yanhe River is the highest in the 1960s; after that time, it shows fluctuating changes. Variation coefficient of runoff is higher than that of precipitation, indicating that the change of runoff is greater than that of precipitation.

Precipitations of different decades in Huangfuchuan are all lower than Yanhe, except in the 1950s; the difference values are all over 100 mm, indicating that the distribution of precipitation in Hekou-Longmen region is greatly different from south to north. Runoff changes in Huangfuchuan are much higher than in Yanhe, for the reason that Yanhe has more precipitation, better surface vegetation, higher soil organic matter, and fine particulate matter [42], which can improve permeability, roughness, and corrosion resistance of the land surface. Thus, changes of runoff in Yanhe fluctuated less than in Huangfuchuan.

MK test is applied to further identify the decreasing trend of runoff in the Hekou-Longmen region, Huangfuchuan, and Yanhe (Table 2). It is calculated that the runoff rank correlation coefficient is −6.18 in the Hekou-Longmen region, −5.18 in Huangfuchuan, and −2.61 in Yanhe, all reaching the significance level 0.01 and the Kendall slopes are all negative. Thus, it can be concluded that, as time goes on, runoff changes in different stations all present a decreasing trend. Runoff in the Hekou-Longmen region is decreasing at an average of −1.0567 per year, which is the most obvious one, and it reached 84.2 × 10^8^ m^3^ in the 1950s and reduced to 23.0 × 10^8^ m^3^ in 2000, and it decreased by 72.7%. Runoff changes in Huangfuchuan and Yanhe have weak decreasing trends with annual decline rate of −0.0305 × 10^8^ m^3^ in Huangfuchuan and −0.0127 × 10^8^ m^3^ in Yanhe. Different from the runoff change, the precipitation shows no obvious decreasing trend; the relation between precipitation and runoff appears inconsistency. It may be influenced by human activities such as urbanization construction, hydraulic projects, and ecological restoration which have changed the underlying surface of the watershed, which caused the change of precipitation-runoff relationship.

### 4.2. The Abrupt Change Points of the Runoff Change

**Figure 3:**
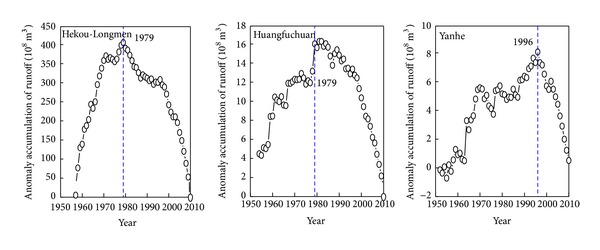
Accumulative anomaly curve of runoff in the Hekou-Longmen region, Huangfuchuan, and Yanhe.

**Figure 4:**
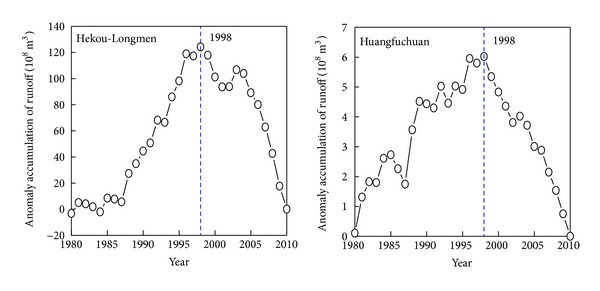
Accumulative anomaly curve of runoff in the Hekou-Longmen region and Huangfuchuan during 1980–2010.

Runoff variation can reflect not only the changing process of precipitation but also reflect the continuous impact of human activities [37]. Anomaly accumulation method is applied to discriminate the abrupt change points of runoff change [43]. The results show that runoffs in the Hekou-Longmen region, Huangfuchuan, and Yanhe show an obvious trend of rising and decreasing (Figure 3). Around 1979, accumulated anomalies of runoff in the Hekou-Longmen region and Huangfuchuan both increase first and then decrease; it can be concluded that the abrupt change point is in 1979. After 1979, the slope of anomaly accumulation of runoff in the Hekou-Longmen region and Huangfuchuan changed in the period 1990–2000, indicating that the precipitation-runoff relationship may have changed. Therefore, anomaly accumulation of runoff from 1980 to 2010 is calculated in the Hekou-Longmen region and Huangfuchuan (Figure 4). It shows that the accumulated anomalies both increase first and then decrease and the abrupt change point is in 1998. Thus, there are two abrupt change points in the Hekou-Longmen region and Huangfuchuan, that is, 1979 and 1998.

Before the 1980s, the influence of human activities on Hekou-Longmen region and Huangfuchuan was relatively small, so, before the abrupt change point in 1979, the runoff was mainly influenced by precipitation. But after 1979, the influence of human activities gradually increased, especially after 1998. Hekou-Longmen region is the mainly sediment source area of the Yellow River and the soil and water loss is serious there. At the beginning of the 1980s, soil and water conservation work has been effectively implemented and accelerated its treatment progress [44]. Take Huangfuchuan as an example; its harnessed degree was only 6.8% before the 1970s, and its forest and grass land made up 86.25% of its control area, and also other engineering measures only accounted for 13.75%. During the 1980s, its harnessed degree had reached to 17.1% in the late 1989 and 28.2% in the late 1997 [45]. Recently, soil and water conservation, ecological restoration, and closed treatment have been carried out successively. With the implement of these policies, harnessed degree of the soil and water conservation such as returning cropland to forest and grass and construction of check dams is increasing gradually and human activities such as coal mining, transportation facilities, and water resources development and utilization also increased significantly.

Different from Hekou-Longmen region and Huangfuchuan, the runoff abrupt change point in Yanhe was found in 1996. At the end of the 20th century, a large scale of ecological restoration projects, such as returning cropland to forest and grass, had reduced the runoff significantly [46]. So the period before 1996 could be regarded as the stage when human activities had no influence on runoff in the Yanhe in this study.

### 4.3. Relationship between Precipitation and Runoff

Precipitation elasticity of runoff can calculate the influence of runoff to precipitation change without the influence of human activities. According to the abrupt change points divided in the previous section, precipitation elasticity of runoff indexes are 1.11 in the Hekou-Longmen region, 1.09 in Huangfuchuan, and 1.26 in Yanhe; that is to say, when the precipitation changes 10%, the runoff changes 11.1%, 10.7%, and 12.6%, respectively (Table 3). During the period affected by human activities, precipitation elasticity of runoff is lower than that in the period without influence of human activities, which shows that runoff change with precipitation in the Hekou-Longmen region and Yanhe is resisted for the reason of human activities including the large-scale soil and water conservation and hydraulic engineering construction; thus, the sensitivity of runoff to the precipitation is weakened. However, the situation in Huangfuchuan is different; in the period influenced by human activities, precipitation elasticity of runoff is much higher than that without influence of human activities. Since 1980, precipitation fluctuation is relatively smaller (*C*_*v*_ = 0.2), while the runoff fluctuation is much larger (*C*_*v*_ = 0.8). Calculated by formula (1), change rates of precipitation and runoff had a big difference, which caused a higher result. Thus, it proved that the formula can only be adopted in the period without human influence (or less influence) or else the result would be much higher than the true value with great influence by human activities.

Precipitation elasticity of runoff in Huangfuchuan (1.09) is lower than in Yanhe (1.26); this is because Huangfuchuan is located in the north and Yanhe in the south of Hekou-Longmen region, and their precipitation distribution and geomorphological types are very different. Huangfuchuan is the rainstorm center of the Hekou-Longmen region, and most of the rainstorms fall in Nalinchuang which is the mainstream of Huangfuchuan. The rainstorm center is often located in the northwest of the basin. The basin land has the characteristics of bare base rock, weak surface permeability, and low storage capacity that make it easy to generate surface runoff. Ran et al. [45] showed in Huangfuchuan that the precipitation amount in flood period accounts for 80% of annual mean precipitation and runoff in flood period accounts for 82.8% of annual runoff. The change rates of precipitation and runoff are relatively consistent. However, the precipitation amount in flood period in Yanhe accounts for 81.2%, but runoff accounts for 68.6%. The reason for the precipitation elasticity of runoff in Yanhe being higher than that in Huangfuchuan is that Yanhe is located in a loess hilly-gully region, where loess voidage ratio is higher and steady infiltration speed is faster, so the runoff coefficient is lower.

The precipitation elasticity of runoff obtained without the influence of human activities can be used to calculate the runoff change caused by precipitation change:

(3) $$\begin{array}{c} \Delta R=\frac{\Delta P}{\bar{P}}\cdot \varepsilon_{p}\cdot \bar{R}, \end{array}$$

where Δ*R* is the change value (10^8^ m^3^) of runoff caused by precipitation change, $\bar{P}$ and $\bar{R}$ are the average precipitation (mm) and runoff (10^8^ m^3^) without the influence of human activities, and Δ*P* is the change value (mm) of precipitation influenced by human activities.

Precipitation and runoff variation in the period influenced by human activities (Figure 4) shows that, compared with the period before the abrupt change point in Hekou-Longmen region, precipitation in early stage of human activities influence (1980–1998) decreased by 10.0% while the runoff decreased by 37.4%. However, precipitation with the influence of intensive human activities (1999–2010) decreased by 13.5%, but runoff decreased by 63.7%. The sharp decrease of precipitation in Hekou-Longmen region shows that the precipitation-runoff relationship has changed significantly. Compared with the period before the abrupt change point, when the precipitation decreased by 9.5%, the runoff decreased by 57.9% in Huangfuchuan during 1980–2010. The runoff decreased by 33.0% when the precipitation decreased by 8.3% in Yanhe. Therefore, it can be concluded that the decrease of runoff is not influenced by precipitation only.

The contribution rates of precipitation to runoff in the period influenced by human activities were calculated with the formula (3). They are 29.7% in Hekou-Longmen region, 17.9% in Huangfuchuan, and 31.6% in Yanhe, respectively (Table 4). By the method of double mass curve, Mu et al. [47] calculated the influence rates of soil and water conservation measures, precipitation, and other human activities on runoff in Hekou-Longmen region during 1952–2009, which were 39%, 29%, and 32%, respectively. Adopting the slope changing ratio of cumulative quantity method, Wang et al. [48] obtained the contribution rates of precipitation to the decrease of runoff in Huangfuchuan, which were 36.43% during 1980–1997 and 16.8l% during 1997–2008. Qiu et al. [46] analyzed the contribution rates of precipitation to the decrease of runoff in Yanhe River during the period of 1952–2008 by the method of double mass curve and the result was 46.2%. All these research results are basically coincided with the results calculated with the method of precipitation elasticity of runoff in this study; thus it can be used as references for the contribution rates of precipitation to runoff.

## 5. Conclusions

This study applied Mann-Kendall test and anomaly accumulation method to analyze the temporal trends and abrupt change point of the runoff and the relationships between precipitation and runoff in the Hekou-Longmen region, Huangfuchuan, and Yanhe. Furthermore, using climate elasticity of runoff method, the causes of the changes of runoff were deduced. The results can be summarized as follows.

1. The precipitation in the Hekou-Longmen region, Huangfuchuan, and Yanhe has no remarkable decreasing trend; runoff appears a significantly decreasing trend (*P* = 0.01) and the annual decreasing rates are −1.0567 × 10^8^ m^3^, −0.0305 × 10^8^ m^3^, and  −0.0127 × 10^8^ m^3^.
2. The abrupt change points of runoff in Hekou-Longmen region and Huangfuchuan are in the years of 1979 and 1998. The period before 1979 has no influence of human activities and the other two periods (1980–1998 and 1999–2010) are in the intensive influence of human activities. The abrupt change point in Yanhe is in the year of 1996. Since the 1980s, the implementation of soil and water conservation, ecological restoration, and the construction of check dams engineering successively have strengthened the water-holding capacity of the basin, which consequently caused the different decreasing characteristics in runoff change.
3. Precipitation elasticities of runoff in Hekou-Longmen region, Huangfuchuan, and Yanhe are 1.11, 1.09, and 1.26, respectively. That is to say, when the precipitation changes 10%, the runoff changes 11.1%, 10.7%, and 12.6%, respectively. By using the precipitation elasticity of runoff without the influence of human activities, contribution rates of precipitation to the runoff in the influence of human activities are calculated, which are 26.4%, 17.9%, and 31.6%, respectively. Precipitation elasticity of runoff method is a relatively simple way that can be used to calculate the contribution rate of climate change to runoff.

This study has quantitatively analyzed the influence of precipitation on runoff with precipitation elasticity of runoff method, but has no consideration on other impacts of factors such as evaporation and temperature, so we need to do a further study to improve the research.

## Figures and Tables

**Table 1:** Characteristics of precipitation and runoff in different decades in the Hekou-Longmen region, Huangfuchuan, and Yanhe.

Year	Hekou-Longmen region				Huangfuchuan				Yanhe			
	Precipitation		Runoff		Precipitation		Runoff		Precipitation		Runoff	
	Average (mm)	*C*_*v*_	Average (10^8^ m^3^)	*C*_*v*_	Average (mm)	*C*_*v*_	Average (10^8^ m^3^)	*C*_*v*_	Average (mm)	*C*_*v*_	Average (10^8^ m^3^)	*C*_*v*_
1950s	506.9	0.25	84.2	0.33	431.7	0.58	2.65	0.64	495.3	0.18	2.14	0.35
1960–1969	501.1	0.26	69.0	0.26	339.5	0.42	1.72	0.63	563.5	0.22	2.48	0.44
1970–1979	439.9	0.19	54.0	0.21	373.4	0.24	1.76	0.62	498.4	0.18	2.06	0.35
1980–1989	433.5	0.15	37.2	0.22	340.9	0.21	1.27	0.59	502.1	0.18	2.08	0.26
1990–1999	413.2	0.15	42.0	0.22	348.2	0.18	0.90	0.61	466.9	0.23	2.08	0.27
2000–2010	424.1	0.14	23.0	0.47	360.1	0.21	0.33	0.92	497.6	0.16	1.45	0.31
Average	**446.7**	**0.20**	**46.8**	**0.47**	**364.3**	**0.32**	**1.34**	**0.84**	**504.1**	**0.19**	**2.03**	**0.37**

**Table 2:** Trend of precipitation and runoff in the Hekou-Longmen region, Huangfuchuan, and Yanhe.

Study area	Precipitation		Runoff	
	*Z* value	*β*	*Z* value	*β*
Hekou-Longmen region	−1.77	−1.5207	−6.18	−1.0567
Huangfuchuan	−0.18	−0.2636	−5.18	−0.0305
Yanhe	−0.40	−0.3335	−2.61	−0.0127

**Table 3:** Estimation of precipitation elasticity of runoff in the Hekou-Longmen region, Huangfuchuan, and Yanhe.

Study area	Periods	Precipitation elasticity of runoff
Hekou-Longmen region	Before 1979	1.11
	1980–1998	0.60
	1999–2010	0.04
	1980–2010	0.40

Huangfuchuan	Before 1979	1.09
	1980–1998	2.78
	1999–2010	2.27
	1980–2010	2.89

Yanhe	Before 1996	1.26
	1997–2010	0.42

**Table 4:** Influence of precipitation on runoff decline in the Hekou-Longmen region, Huangfuchuan, and Yanhe.

Study area	Periods	Precipitation (mm)	Runoff (10^8^ m^3^)	Changes in precipitation (%)	Changes in runoff (%)	Effect of precipitation on runoff decline	
						Amount (10^8^ m^3^)	Percentage %
Hekou-Longmen region	Before 1979	477.8	64.4	—	—		
	1980–1998	430.0	40.3	10.0	37.4	7.15	26.4%
	1999–2010	413.5	23.4	13.5	63.7	9.61	23.5%
	1980–2010	423.6	33.7	11.3	47.7	8.10	29.7%

Huangfuchuan	Before 1979	384.2	1.95	—	—		
	1980–1998	348.9	1.14	9.2	41.5	0.195	24.1%
	1999–2010	345.6	0.32	10.0	83.6	0.213	13.1%
	1980–2010	347.6	0.82	9.5	57.9	0.202	17.9%

Yanhe	Before 1996	514.3	2.21	—	—		
	1997–2010	471.7	1.48	8.3	33.0	0.231	31.6%
